# A Comparative Proteomic Analysis of Extracellular Vesicles Associated With Lipotoxicity

**DOI:** 10.3389/fcell.2021.735001

**Published:** 2021-11-04

**Authors:** Yasuhiko Nakao, Masanori Fukushima, Amy S. Mauer, Chieh-Yu Liao, Anya Ferris, Debanjali Dasgupta, Carrie Jo Heppelmann, Patrick M. Vanderboom, Mayank Saraswat, Akhilesh Pandey, K. Sreekumaran Nair, Alina M. Allen, Kazuhiko Nakao, Harmeet Malhi

**Affiliations:** ^1^ Division of Gastroenterology and Hepatology, Rochester, MN, United States; ^2^ Department of Gastroenterology and Hepatology, Graduate School of Biomedical Sciences, Nagasaki University, Nagasaki, Japan; ^3^ California Polytechnic State University, San Luis Obispo, CA, United States; ^4^ Department of Physiology and Biomedical Engineering, Manipal, India; ^5^ Mayo Clinic Medical Genome Facility-Proteomics Core, Manipal, India; ^6^ Mayo Endocrine Research Unit, Manipal, India; ^7^ Department of Laboratory Medicine and Pathology, Rochester, MN, United States; ^8^ Institute of Bioinformatics, Bangalore, India; ^9^ Manipal Academy of Higher Education (MAHE), Manipal, India; ^10^ Center for Individualized Medicine, Rochester, MN, United States

**Keywords:** exosome, microvesicle, DAMP, hepatocyte, StAR-related lipid transfer domain 11

## Abstract

Extracellular vesicles (EVs) are emerging mediators of intercellular communication in nonalcoholic steatohepatitis (NASH). Palmitate, a lipotoxic saturated fatty acid, activates hepatocellular endoplasmic reticulum stress, which has been demonstrated to be important in NASH pathogenesis, including in the release of EVs. We have previously demonstrated that the release of palmitate-stimulated EVs is dependent on the *de novo* synthesis of ceramide, which is trafficked by the ceramide transport protein, STARD11. The trafficking of ceramide is a critical step in the release of lipotoxic EVs, as cells deficient in STARD11 do not release palmitate-stimulated EVs. Here, we examined the hypothesis that protein cargoes are trafficked to lipotoxic EVs in a ceramide-dependent manner. We performed quantitative proteomic analysis of palmitate-stimulated EVs in control and STARD11 knockout hepatocyte cell lines. Proteomics was performed on EVs isolated by size exclusion chromatography, ultracentrifugation, and density gradient separation, and EV proteins were measured by mass spectrometry. We also performed human EV proteomics from a control and a NASH plasma sample, for comparative analyses with hepatocyte-derived lipotoxic EVs. Size exclusion chromatography yielded most unique EV proteins. Ceramide-dependent lipotoxic EVs contain damage-associated molecular patterns and adhesion molecules. Haptoglobin, vascular non-inflammatory molecule-1, and insulin-like growth factor-binding protein complex acid labile subunit were commonly detected in NASH and hepatocyte-derived ceramide-dependent EVs. Lipotoxic EV proteomics provides novel candidate proteins to investigate in NASH pathogenesis and as diagnostic biomarkers for hepatocyte-derived EVs in NASH patients.

## Introduction

Extracellular vesicles (EVs) are emerging mediators of liver injury and inflammation in nonalcoholic steatohepatitis (NASH), a lipotoxic disorder characterized by the accumulation of the toxic saturated fatty acid, palmitate (Hirsova et al., 2016a; Kakazu et al., 2016). EVs are heterogeneous membrane-defined nanoparticles released by most cell types. The quantity and cargo of EVs shift from healthy to disease states and reflects the pathophysiological state of the cell of origin (Goetzl et al., 2016; Sehrawat et al., 2020; Nakao et al., 2021). Furthermore, the mechanisms leading to the formation and release of EVs and of cargo selection in to EVs remain incompletely understood (van Niel et al., 2018). We have previously demonstrated that palmitate-induced activation of the endoplasmic reticulum (ER) stress sensor inositol requiring enzyme 1 alpha (IRE1α) mediates the release of EVs *via* the *de novo* synthesis of ceramide (Fukushima et al., 2018). These lipotoxic EVs are enriched in ceramides and other sphingolipids (Kakazu et al., 2016; Dasgupta et al., 2020). In addition to lipid cargoes, EVs contain protein and nucleic acid cargoes (Povero et al., 2014a). However, whether protein selection or trafficking into lipotoxic EVs is impacted by ceramide trafficking remains unknown.

There are no gold standard methods for EV isolation, and each method isolates EVs with variable purity and yield (Li et al., 2019). EVs have broad biophysical properties and many methods of EV isolation rely on these biophysical properties or on protein antigens for antibody-based EV isolation. For proteomic studies, differential ultracentrifugation (UTC) is one of the most utilized EV isolation techniques (Serrano et al., 2012; Mathieu et al., 2019), though increasingly size exclusion chromatography (SEC) and density gradient (DG) flotation (Kowal et al., 2016) are being employed. Here, we initially compared EV proteomics data obtained from mass spectrometry of EVs isolated by utilizing these three methods. Based on our initial results, we next utilized SEC for comparative proteomics of EVs isolated from palmitate-treated hepatocyte cell lines from cells with intact or deficient ceramide trafficking (Fukushima et al., 2018). In addition, we performed human plasma EV proteomics, where EVs were isolated by SEC or DG from control and NASH plasma samples, for comparison with the proteome of hepatocyte-derived EVs.

Our proteomics data demonstrate that SEC yielded most unique proteins, followed by UTC, and then DG (Keerthikumar et al., 2016; Consortium, 2020). Lipotoxic EVs collected by SEC contained numerous ribosomal proteins. Further analysis demonstrated that ceramide-dependent lipotoxic EVs contained damage-associated molecular patterns (DAMPs) and cell adhesion molecules. Human NASH EVs included immune response processing proteins. Haptoglobin, vanin 1 (VNN1), and insulin-like growth factor-binding protein complex acid labile submit (IGFALS) were commonly detected in NASH EVs and hepatocyte-derived ceramide-dependent EVs.

## Materials and Methods

**Cells**. We used previously described mouse hepatocyte cell lines derived from Immortalized Mouse Hepatocytes (IMH) and CRISPR/Cas9-mediated deletion of STARD11 (STARD11-/-) in IMH cells, and empty vector controls [designated throughout this manuscript as wild type (WT)] (Fukushima et al., 2018). Cells were cultured to 90% confluency on 150-mm tissue culture dishes. Cells were washed twice with phosphate-buffered saline (PBS) to eliminate fetal bovine serum (FBS)-derived EVs. Then, cells were treated with either 400 µM palmitate or vehicle in Dulbecco’s Modified Eagle Medium (DMEM) supplemented with 5% EV-depleted FBS, 1% bovine serum albumin (BSA), and penicillin and streptomycin for 16 h (Fukushima et al., 2018). EV-depleted FBS was prepared by overnight centrifugation at 100,000 × g at 4°C according to standard protocols (Théry et al., 2006). Conditioned medium from palmitate or vehicle-treated cells was collected before the onset of apoptosis using a validated concentration and duration (Kakazu et al., 2016; Fukushima et al., 2018). Collected cell-conditioned medium was depleted of cells and cellular debris initially by low-speed centrifugation at 2000 × g for 20 min and 10,000 × g for 40 min. Small EVs were next isolated as described below. For each experimental condition, isolated EVs were normalized to cell number and expressed relative to the vehicle-treated condition.

**Size exclusion chromatography.** The supernatant of the 10,000 × g spin was concentrated by a centrifugal filter device (Centricon Plus-70) at 3,500 × g for 40 min. After the concentration step, the device was inverted and centrifuged at 1,000 × g for 2 min to recover the concentrated supernatant. The concentrated supernatants were next diluted with PBS to a total volume of 2 ml. These were then applied to primed 2-ml size exclusion columns (qEV2, IZON, Medford, MA), followed by elution with PBS (pH 7.4, 0.22 µm filtered). The first void volume (13 ml, fractions 1–6.5) was discarded and fractions 6.5–10.5 were collected and pooled, per the manufacturer’s instructions. The EVs in pooled fractions 6.5–10.5 were pelleted by UTC at 100,000 × g for 2 h and resuspended in 100 μL of PBS.

**Differential ultracentrifugation.** The supernatant of the 10,000 × g spin was followed by UTC at 100,000 × g for 90 min to pellet EVs. The pellets were washed in PBS and centrifuged again 100,000 × g for 2 h and resuspended in 100 μL of PBS.

**Differential ultracentrifugation followed by iodixanol density gradient separation.** Iodixanol density gradient separation was performed (Fukushima et al., 2018) and collected fractions were combined as follows: fractions 1–2, fractions 3–6, and fractions 7–10, respectively. Each combined fraction was centrifuged at 100,000 × g for 2 h and resuspended in 100 μL of PBS. Five microliters was reserved for nanoparticle tracking analysis (NTA), and the remainder was employed for quantitative proteomic analysis. The density of each fraction was confirmed with a refractometer (Supplementary Figure S1). This method was based on the protocol described by Kowal et al. (Kowal et al., 2016).

**Plasma EV isolation.** Banked EDTA fasting plasma was obtained from adults following written informed consent using materials approved by the Mayo Clinic’s Institutional Review Board. We pooled plasma from two males with NASH diagnosed on expert pathologist review of liver biopsy and divided the plasma into 1-ml aliquots each for SEC and DG. The control plasma was from an age- and sex-matched individual with no underlying liver disease. Plasma samples were thawed on ice and divided into two sets of 1 ml each for SEC and DG, respectively. Each plasma aliquot was centrifuged at 3,000 × g for 15 min at room temperature to obtain clarified supernatants. Nine hundred microliters of supernatant was transferred to new tubes and diluted 1:1 with PBS, pH 7.4, and centrifuged at 2,000 × g for 30 min at 4°C. The resulting supernatant was then centrifuged at 12,000 × g for 45 min at 4°C. For SEC, this supernatant was applied to primed 2-ml size exclusion columns as described above. For DG, the supernatant was adjusted to 10 ml with PBS and centrifuged at 110,000 × g for 120 min to pellet EVs, which were applied to the iodixanol density gradient as described above.

**Nanoparticle tracking analysis.** Size distribution and concentration of isolated EVs was assessed by NTA as previously described by us (Kakazu et al., 2016). Briefly, EVs were diluted in PBS to be in the linear dynamic range of the instrument and each sample was perfused through the chamber at a rate of 25 μL/min while recording the Brownian motion of particles. Particle tracks were analyzed to measure the concentration of the particles (particles/ml) and size (in nanometers).

**Quantitative proteomics analysis.** This was performed at the Mayo Clinic Proteomics Core Laboratory. For the method finding studies, one biological replicate each was included. Three biological replicates were included for the comparative proteomics in WT and STARD11-/- cell with palmitate or vehicle treatment. For sample preparation, EVs were lysed in 50 mM Tris containing 0.1% SDS. Samples were vortexed and heated to 95°C followed by snap cooling on ice. Tris (2-corboxyethyl)phosphine hydrochloride solution (Sigma) was added to a final concentration of 10 mM and incubated at 65°C for 30 min followed by the addition of iodoacetamide (Sigma) to a final concentration of 20 mM and incubated at room temperature in the dark for 30 min. Digestion of the EV proteins was accomplished by adding 0.6 µg of trypsin (Promega) and incubated at 37°C overnight. Sample clean-up was performed using Detergent Removal Spin Columns (Pierce), according to directions. EV peptide samples were analyzed by nano-flow liquid chromatography electrospray tandem mass spectrometry (nanoLC-ESI-MS/MS) using a Thermo Scientific Q-Exactive Mass Spectrometer (Thermo Fisher Scientific, Bremen, Germany) coupled to a Thermo Ultimate 3,000 RSLCnano HPLC system. Sample digests were loaded onto a 0.33-µL PEP ES-C18 trap, 330-nL Halo 2.7 ES-C18 trap (Optimize Technologies, Oregon City, OR). Chromatography was performed using a 5–45% gradient of solvent B over 90 min where solvent A is (98% water/2% acetonitrile/0.2% formic acid) and solvent B is (80% acetonitrile/10% isopropanol/10% water/0.2% formic acid). Peptides were eluted at a flow rate of 400 nL/min from the trap through a PicoFrit (New Objective, Woburn, MA) 100 μm × 33 cm column hand packed with Agilent Poroshell 120 EC C18 packing (Agilent Technologies, Santa Clara, CA). Q-Exactive mass spectrometer was set to acquire an ms1 survey scan from 350 to 1,600 m/z at resolution 70,000 (at 200 m/z) with an AGC target of 3e6 ions and a maximum ion inject time of 60 ms. Survey scans were followed by HCD MS/MS scans on the top 15 ions at resolution 17,500, AGC target of 2e5 ions, maximum ion inject time of 60 ms, and the isolation window set at 2.5 m/z with a 0.3 m/z offset. Dynamic exclusion placed selected ions on an exclusion list for 40 s.

**Method determination:** In-house software was used to set up database searches in Mascot (Matrix Sciences) and X!Tandem (https://www.thegpm.org/tandem/). Scaffold (Proteome Software) was used to combine and view these results. Criteria for protein identification required two peptide minimum and a protein 0.5% false discovery rate (FDR). Total spectral count was used as a rough estimation of protein concentration. To compare across groups (*n* = 3) the MaxQuant software (Max Planck Institute of Biochemistry, Martinsried, Germany), version 1.6.0.16, was used to extract, time align, and database search chromatographic extracted peptide peaks generated from mass spectrometry files (first and second MQ reference). Label-free relative quantitation parameters within the MaxQuant software were used to generate normalized protein intensities reported in a protein groups table (third MQ reference). Perseus software (Max Planck Institute of Biochemistry, Martinsried, Germany), version 1.6.2.1, was used to perform differential expression of identified proteins (Perseus reference). Briefly, protein intensities were log2 transformed, missing values were imputed, Student’s *t*-tests were performed in which an estimation of difference was calculated, and *p*-values and *q*-values were reported.

**Statistical and data analysis.** All analyses, except the ones reported above, and graphical preparations were conducted in GraphPad Prism 8 (GraphPad Software Inc., La Jolla, CA). Figures were prepared with R-studio and the package ggplot2, heatmaply, and Rtsne. For data analysis of EV proteomics, ExoCarta (www.ExoCarta.org) (Keerthikumar et al., 2016), MetaboAnalyst (www.metaboanalyst.ca) (Pang et al., 2021), FUNRICHNEW (www.funrich.org) (Benito-Martin and Peinado, 2015), STRING (www.string-db.org) (Szklarczyk et al., 2019), and Ingenuity (QIAGEN Inc., https://www.qiagenbioinformatics.com/products/ingenuitypathway-analysis) Pathway Analysis (Krämer et al., 2013) were used. FDR was calculated by Benjamini–Hochberg procedure.

## Results

**FIGURE 1:**
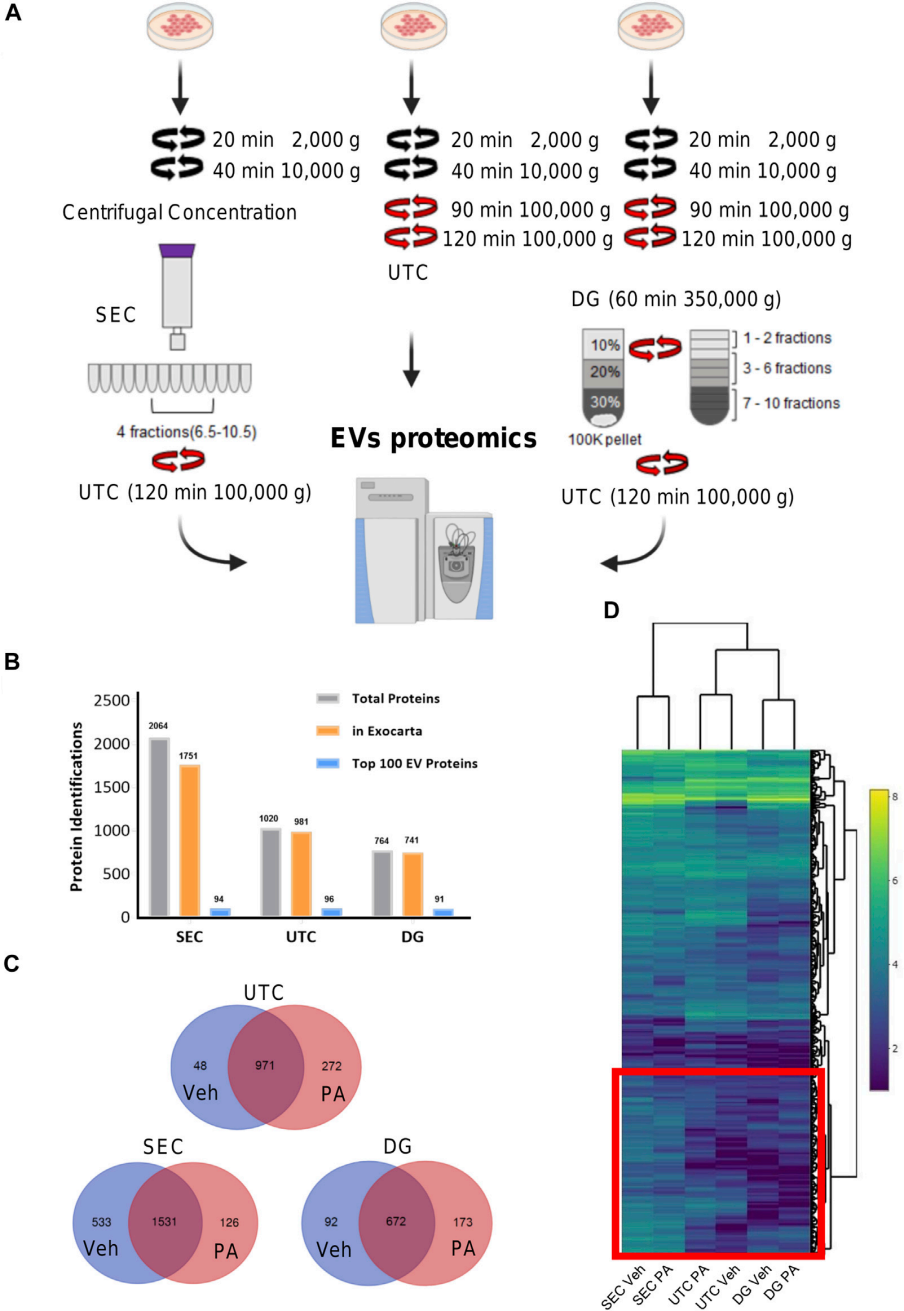
Overview of EV proteomics methods. **(A)** Schema represents three extracellular vesicle (EV) isolation methods for EV proteomics. In size exclusion chromatography (SEC) methods, fractions 6.5 to 10.5 were combined and pelleted by ultracentrifugation (UTC). For the UTC sample, the 100,000 × g fraction was utilized. For Iodixanol, density gradient (DG) fractions 1–2, 3–6, and 7–10 were collected and combined. **(B)** The number of identified proteins for each of the three methods, SEC, UTC, and DG. The gray bars display the total number of identified proteins, the orange bars display the number of the total protein IDs that were found in the ExoCarta database, and the blue bars display the number of proteins from each method that was found in the ExoCarta top 100 EV proteins. **(C)** Venn diagrams depicting the number of unique proteins that were detected in vehicle (Veh) or palmitate (PA) stimulated EVs by each method. **(D)** Heatmap shows common 537 proteins that were detected by each method. Heatmap color represents Log2 protein abundances. The red box encloses the cluster of proteins with higher abundance in EVs isolated by SEC.

**FIGURE 2:**
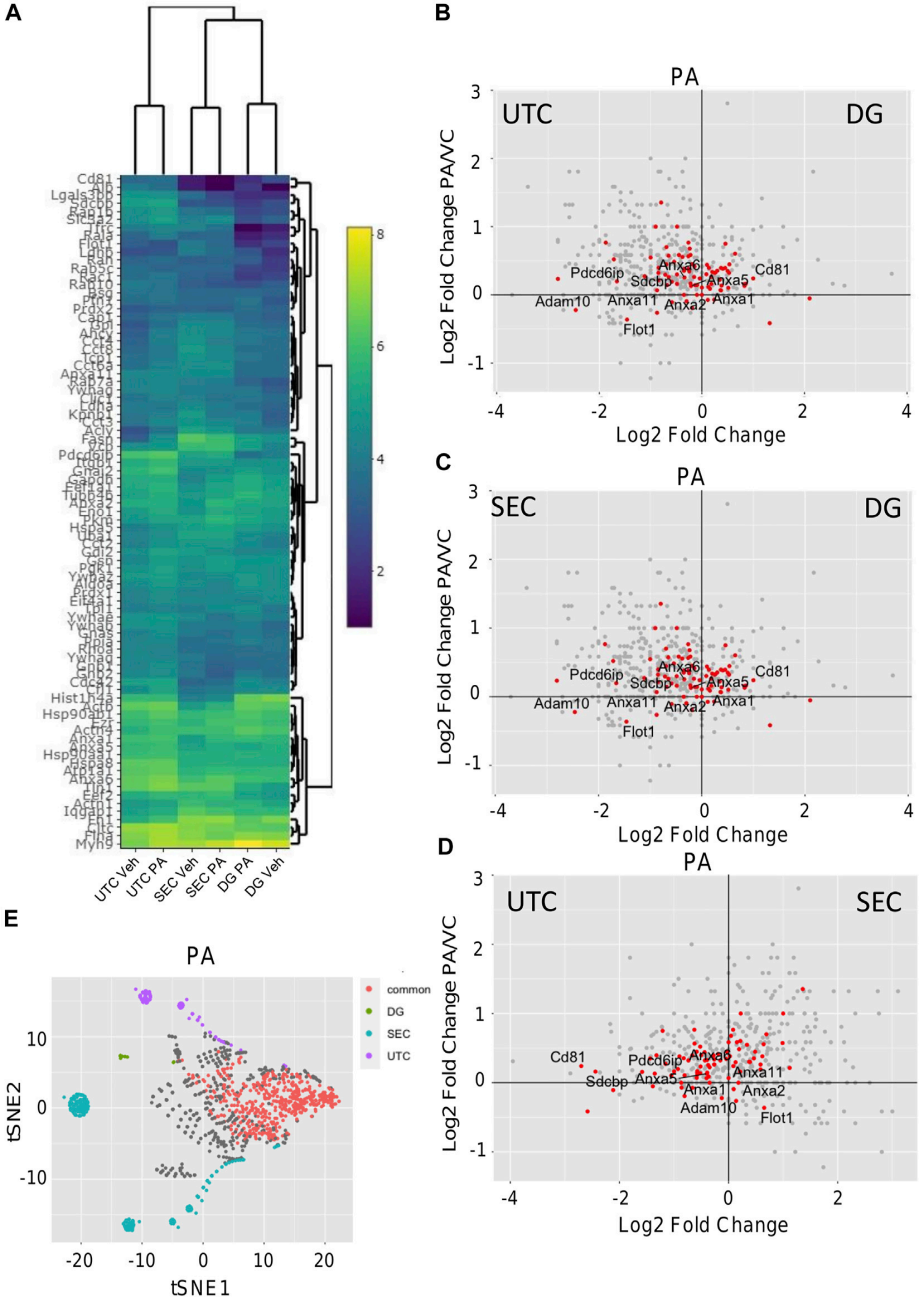
Comparison of EV proteomics. **(A)** Heatmap depicting the abundance 83 common proteins (Log2 protein abundance) of ExoCarta top 100 EV proteins detected by each method. **(B)** Differential expression of proteins by volcano plot where the *x*-axis represents Log2 (DG PA/UTC PA) and the *y*-axis represents Log2 (UTC PA/UTC Veh). **(C)** Differential expression of proteins by volcano plots where the *x*-axis represents Log2 (DG PA/SEC PA) and the y-axis represents Log2 (UTC PA/UTC Veh). **(D)** Differential expression of proteins by volcano plots where the x-axis represents Log2 (SEC PA/UTC PA) and the y-axis represents Log2 (UTC PA/UTC Veh). For **(B–D)**, red dots indicate proteins from the top 100 ExoCarta EV proteins. **(E)** tSNE dimension reduction analysis was performed by Rtsne R package in all the data including missing values from PA stimulated EVs to depict the commonly and uniquely detected proteins by each of the three methods.

**Comparison of EV isolation methods for proteomics.** To investigate the optimal isolation technique for EV proteomics, EVs were isolated from vehicle-treated IMH cells by SEC, UTC, or DG (Figure 1A). Figure 1B displays the number of identified proteins for each method. Each color bar represents total number of identified proteins, the number of the total protein IDs that were found in the ExoCarta database (Keerthikumar et al., 2016), and the number of proteins from each method that were found in the ExoCarta top 100 EV proteins, respectively (Keerthikumar et al., 2016). This showed that SEC yielded the most proteins and almost as many proteins in the ExoCarta database as UTC. The yield of total proteins and ExoCarta proteins was least in DG. The yield of proteins isolated by UTC was in between SEC and DG. Thus, SEC had the highest sensitivity to detect proteins. To determine the specificity of these methods in enriching EV proteins, we determined the yield of top 100 EV proteins across the three methods. Table 1 demonstrates a summary of 14 common EV markers that were detected by each method under basal conditions. All 14 were detected by UTC; however, SEC was non-inferior by detection 13 out of 14 common EV markers, and 94 of top 100 ExoCarta proteins in comparison to 96 detected by UTC. Next, we examined the EV proteome from palmitate-treated IMH cells in comparison to the vehicle-treated cells. Like vehicle-treated EVs, palmitate-stimulated EVs yielded the most proteins by SEC, followed by UTC, followed by DG. The Venn diagrams (Figure 1C) illustrate the comparison of vehicle- and palmitate-stimulated EV proteins for each of the three isolation methods. The Venn diagrams demonstrate that there was a significant shift in the EV proteome following palmitate treatment. To compare the abundance of proteins across methods in vehicle- and palmitate-stimulated EVs, we excluded all proteins that were not detected across all six conditions. A total of 537 proteins were detected in EVs across all six conditions (Figure 1D). In the 537 commonly detected proteins, there was variability across methods and proteins were more abundantly detected by SEC. However, 83 of the top 100 ExoCarta EV proteins that were detected by all methods were of comparable abundance across conditions (Figure 2A). Figures 2B–D represent comparison of palmitate upregulated proteins detected by each method. The abundance of palmitate-stimulated EV proteins detected by each method, indicated in the figures, was compared with palmitate-stimulated EV proteins enriched in EVs isolated by UTC (*y*-axis, Figures 2B–D). Within these plots, the red dots represent EV proteins that are included in the top 100 ExoCarta EV proteins. Comparative analysis of EV proteins enriched in palmitate-stimulated EVs by each method demonstrated higher abundance of EV proteins by UTC than DG (Figure 2B), and higher by SEC than DG (Figure 2C). When comparing UTC to SEC, most EV protein abundances were comparable except CD81 (Figure 2D). Figure 2E represents t-distributed stochastic neighbor embedding (tSNE) analysis of all the data including missing values. The number of unique proteins that were only detected by SEC, UTC, and DG were 797, 140, and 30, respectively. Altogether, SEC detected most unique proteins and was comparable to UTC and DG for the detection of top 100 ExoCarta EV proteins. Thus, SEC had the most sensitivity to detect proteins while retaining specificity for known EV proteins. As our objective was to discover unique palmitate-stimulated ceramide-dependent EV proteins, based on this initial method finding analysis, we pursued SEC for further proteomic analysis of ceramide-dependent proteomic changes in EV cargo.

**TABLE 1:** EV markers identified by SEC, UTC, and DG f3-6.

	SDCBP	ADAM10	PDCD6IP	CD63	CD9	CD81	TSG101	Flot1	Anxa1	Anxa2	Anxa5	Anxa6	Anxa7	Anxa11
SEC	X	X	X	—	X	X	X	X	X	X	X	X	X	X
UTC	X	X	X	X	X	X	X	X	X	X	X	X	X	X
DG f3-6	X	X	X	—	—	X	—	X	X	X	X	X	—	X

**FIGURE 3:**
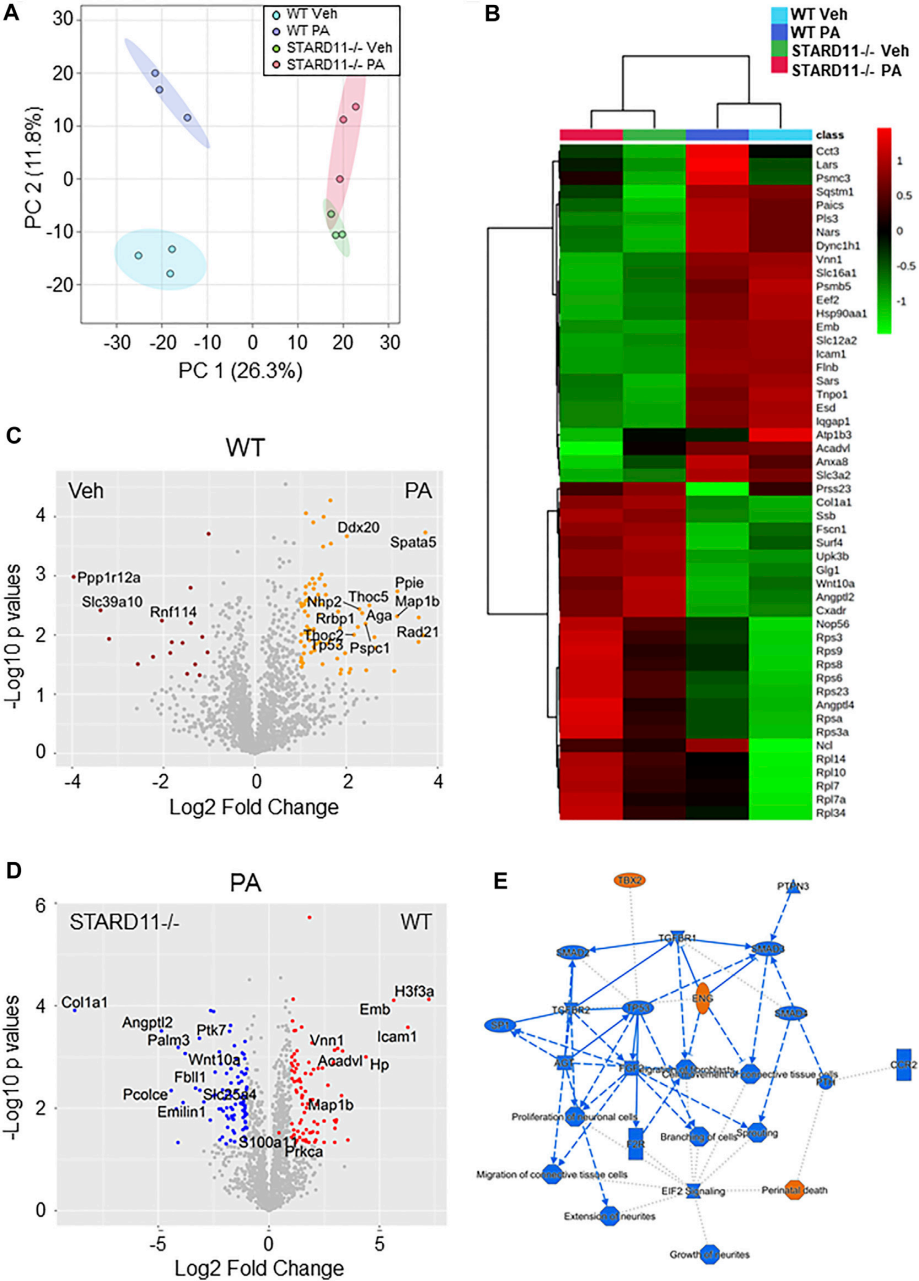
Comparative proteomics of lipotoxic EVs from WT or STARD11-/- cells. **(A)** Principal component analysis of proteins detected in wild-type (WT) or STARD11 knockout (STARD11-/-) extracellular vesicles (EVs) from palmitate (PA)- or vehicle (Veh)-treated cells. **(B)** The heatmap depicts top 50 significant proteins between four different groups. **(C)** Volcano plot shows comparison between WT Veh and WT PA; orange circle represents *p* < 0.05 and Log2 Fold change > 1; red circle represents *p* < 0.05 and Log2 Fold change < −1. **(D)** Volcano plot shows comparison between STARD11-/- PA and WT PA; red circle represents *p* < 0.05 and Log2 Fold change > 1; blue circle represents *p* < 0.05 and Log2 fold change < −1. **(E)** Figure shows ingenuity pathway analysis based on WT PA/STARD11-/- PA data.

**Lipotoxic EV proteins are significantly altered by knockout of StAR-related lipid transfer domain 11 (STARD11).** We have previously demonstrated that *de novo* ceramide biosynthesis and ceramide trafficking by the ceramide transport protein STARD11 mediates the release of lipotoxic EVs (Kakazu et al., 2016; Fukushima et al., 2018). To assess cargoes that may be selected in to lipotoxic EVs in a ceramide-dependent manner, we compared the proteome of equal amounts of lipotoxic EV proteins derived from WT or STARD11-/- cells treated with palmitate. EVs were isolated by SEC and characterized by mass spectrometry. Quantification of EVs isolated from STARD11-/- and WT cells by SEC (Supplementary Figure S2) was consistent with our previous observations that STARD11 mediates the release of palmitate-stimulated EVs (Fukushima et al., 2018). A Venn diagram of proteins identified in EVs from WT or STARD11-/- cells with or without palmitate treatment (Supplementary Figure S3) demonstrated that 1,146 proteins were commonly detected in all four conditions. To try to understand relational differences between the samples, we performed principal component analysis of each condition (Figure 3A). We observed that the proteome of WT EVs was different from STARD11-/- EVs both basally and following palmitate treatment. Palmitate treatment led to a shift in the proteome of EVs in both the WT control cell and STARD11-/- cells. A heatmap of the 50 most significantly different proteins from the four conditions (Figure 3B) demonstrates that proteins significantly enriched in STARD11-/- EVs were depleted in WT EVs, and *vice versa*, suggesting that ceramide trafficking influences the EV proteome. To understand the role of palmitate and ceramide trafficking in EV abundance of specific cargoes, we compared the magnitude of change and *p*-values. The volcano plot (Figure 3C) depicts the most significantly palmitate-regulated EV proteins. Spermatogenesis-associated protein 5 (SPATA5) was the most significantly upregulated protein in palmitate-stimulated EVs. The comparison between palmitate-stimulated EVs from WT versus STARD11-/- cells (Figure 3D) demonstrated significant enrichment of histone H3.3 (H3F3A), intercellular adhesion molecule 1 (ICAM1), embigin (Emb), haptoglobin (Hp), immunoglobulin superfamily member 3 (IGSF3) in WT versus STARD11-/- palmitate-stimulated EVs. In contrast, collagen alpha-1 chain (COL1A1), angiopoietin-like protein 2 (ANGPTL2), paralemmin-3 (PALM3), protein tyrosine kinase 7 (PTK7), and Wnt family member 10A (WNT10A) were among the least abundant proteins in palmitate-stimulated lipotoxic EVs, being depleted in WT and enriched in STARD11-/- EV proteins. Based on these data, we performed pathway analysis by IPA software, to investigate STARD11-dependent lipotoxic EV proteins. Graphical summary of the pathway analysis is provided in Figure 3E, and Table 2 lists the top 10 canonical pathways of the 74 that were upregulated. EIF2 signaling was the top canonical pathway upregulated in WT versus STARD11-/- lipotoxic EVs.

**TABLE 2:** Top 10 canonical pathways in WT versus STARD11-/- PA EV proteins.

Ingenuity canonical pathways	−log (*p*-value)
EIF2 signaling	12.1
mTOR signaling	5.32
Coronavirus pathogenesis pathway	4.69
Hepatic fibrosis/Hepatic stellate cell activation	4.5
Regulation of eIF4 and p70S6K Signaling	4.28
Tumor microenvironment pathway	4.05
Actin cytoskeleton signaling	3.68
Tight junction signaling	3.56
Mechanisms of viral Exit from host cells	3.41
NRF2-mediated oxidative stress response	3.16

**FIGURE 4:**
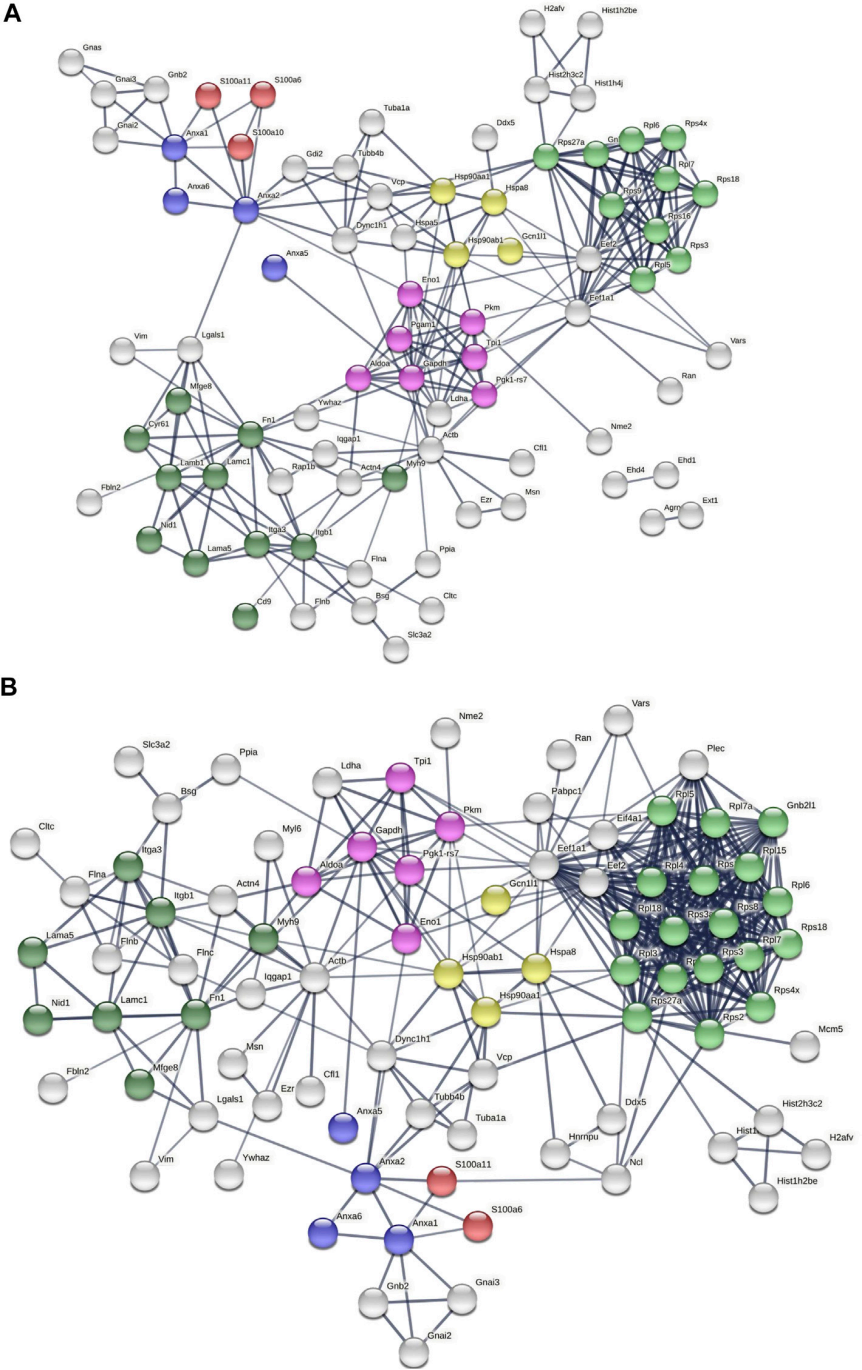
Predicted protein–protein interactions among WT EV proteins. Protein–protein interaction analysis was performed by STRING software **(A)** on WT Veh top 100 expressed proteins, and **(B)** on WT PA top 100 expressed proteins. The edges indicate both functional and physical protein associations; line thickness indicates the strength of data support; we used minimum required interaction score with high confidence (0.700); disconnected nodes in the network were excluded; red color depicts the S100 family proteins; blue color represents the annexin family; green color represents ribosomal proteins; dark green represents cell adhesion proteins; yellow color represents stress response proteins; and pink color represents glycolysis proteins. These proteins were annotated by using UniProt and InterPro.

**FIGURE 5:**
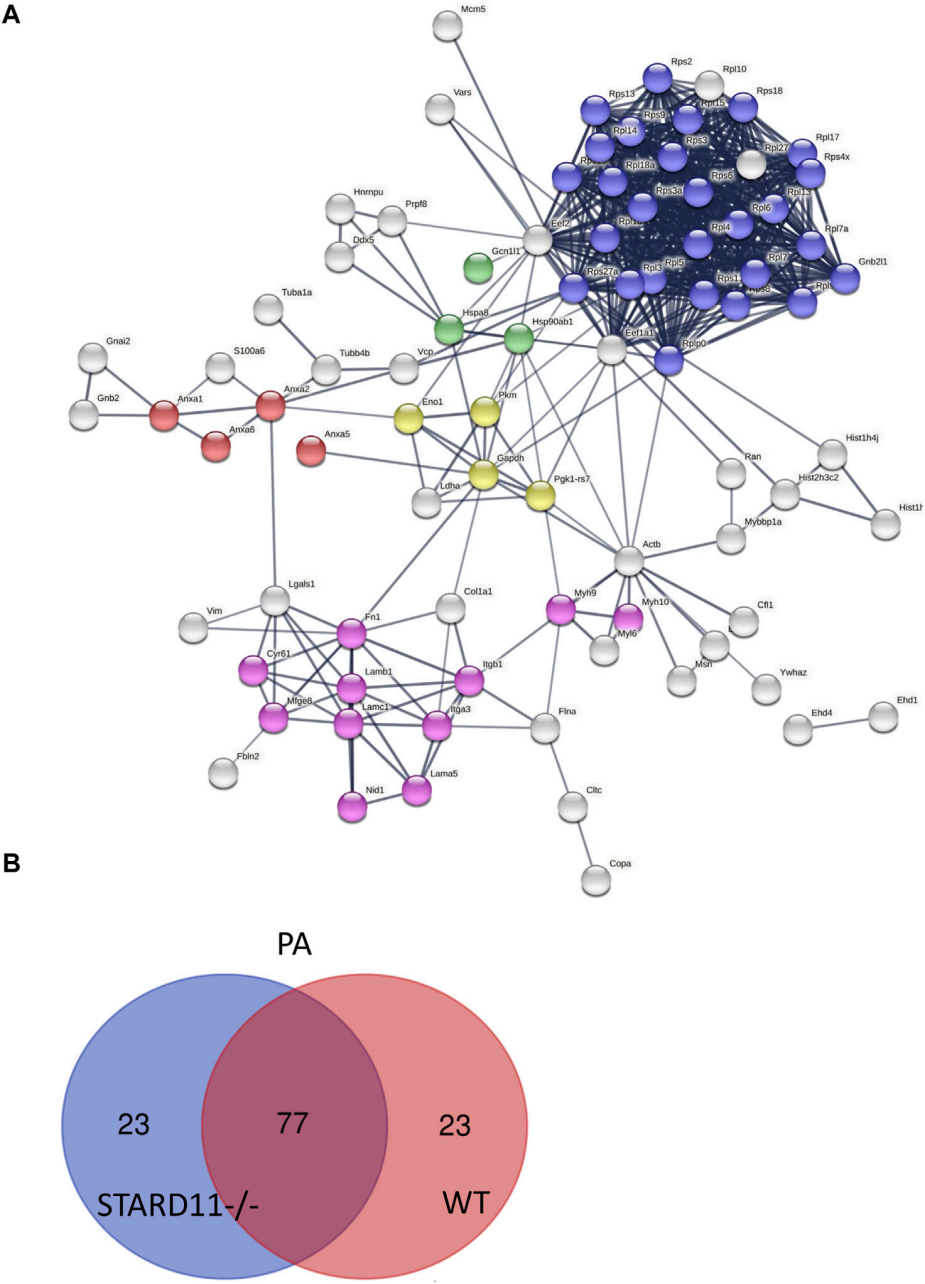
Predicted protein–protein interactions among STARD11-/- EV proteins. **(A)** Protein–protein interaction analysis was performed by STRING software on STARD11-/- PA top 100 expressed proteins; red color shows annexin family proteins; blue color represents ribosomal proteins; green color represents stress response proteins; yellow color represents glycolysis proteins; and pink color represents cell adhesion proteins. **(B)** The Venn diagram represents the number of common and unique proteins between STARD11-/- PA and WT PA EV proteins.

We next parsed our EV proteomics data with STRING to gain a functional assessment of EV cargoes and to understand known and predicted protein–protein interactions. We compared the proteomics of vehicle- and palmitate-stimulated EVs in the control cells. We selected the top 100 expressed proteins and analyzed by STRING software to determine protein–protein interactions and functional enrichment (Figure 4A). EV proteins included annexin A1, A2, A5, A6 (ANXA1, ANXA2, ANXA5, and ANXA6), S100 family proteins (A6, A10, and A11), ribosomal proteins, cell adhesion related proteins, stress response proteins (HSP90AA1, HSP90AB1, and GCN1l1), and glycolysis-related proteins. The number of ribosomal proteins were increased in palmitate-stimulated lipotoxic EVs (Figure 4B). Supplementary Figure S4 represents gene ontology (GO) analysis of the most abundant proteins; palmitate-stimulated EVs were enriched in ribosomal functions, RNA binding, protein translation, angiogenesis, and cell division. Next, STRING analysis was performed on the proteomics of STARD11-/- lipotoxic palmitate-stimulated EVs. We observed that ribosomal proteins were also enriched in these EVs (Figure 5), similar to WT palmitate-stimulated EVs. The Venn diagram (Figure 5B) compared the proteome of the top 100 palmitate-stimulated EV proteins in STARD11-/- versus WT. We detected 23 unique proteins each in STARD11-/- and WT EVs, whereas 77 proteins were conserved, and included numerous ribosomal proteins. In WT palmitate-stimulated EVs, some of the unique proteins included S100A11, EIF4A1, and IQGAP1; a complete list of these 23 proteins is provided in Table 3. Among trafficking machinery components, we detected 65 proteins (Table 4). Of these 65 proteins, 10 were significantly different, of which 6 were upregulated and 4 were downregulated. The majority of trafficking proteins (55 proteins) were not significantly different between in WT versus STARD11-/- lipotoxic EVs. Whether the 10 differentially regulated proteins may impact cargo selection remains to be tested.

**TABLE 3:** Ceramide-dependent PA-stimulated EV proteins.

Protein IDs
Ext1	Plec	S100a11	Gmai3
Plxnb2	Ppia	Flnb	Myof
Banf1	Flnc	Hsp90aa1	—
Bsg	Actn4	Aldoa	—
Pabpc1	Shmt2	Iqgap1	—
Nme2	Dync1h1	Ncl	—
Eif4a1	H2afv	Tpi	—

**TABLE 4:** Trafficking proteins in palmitate stimulated EVs.

Protein description	Gene	Log2 fold change WTPA and STARD11-/-PA	*p*-value
Metalloreductase STEAP2	Steap2	1.88903333	0.14282126
Ras-related protein Rab-3B	Rab3b	1.40553333	0.01943646
Vesicle transport through interaction with t-SNAREs homolog 1B	Vti1b	1.1873	0.13940431
COP9 signalosome complex subunit 4	Cops4	1.15356667	0.19666602
Unconventional myosin-Va	Myo5a	0.95773333	0.25756553
Ras-related protein Rab-5B	Rab5b	0.94836667	0.01680058
Ras-related protein Rab-27B	Rab27b	0.9091	0.59200059
Clathrin light chain A	Clta	0.84713333	0.02919789
Caveolin-2	Cav2	0.74893333	0.10698931
Lysosome-associated membrane glycoprotein 1	Lamp1	0.7218	0.14618078
Phosphatidylethanolamine-binding protein 1	Pebp1	0.6983	0.00896184
Phosphatidylinositol 4-kinase type 2-alpha	Pi4k2a	0.64753333	0.03888959
Guanine nucleotide-binding protein G(s) subunit alpha isoforms short	Gnas	0.5608	0.01037145
Vesicle-associated membrane protein 8	Vamp8	0.51643333	0.27127569
Clathrin light chain B	Cltb	0.4653	0.10648827
AP-2 complex subunit alpha-2	Ap2a2	0.3966	0.23324825
Clathrin heavy chain 1	Cltc	0.38223333	0.34105627
PDZ domain-containing protein GIPC1	Gipc1	0.38076667	0.39327463
Cell cycle control protein 50A	Tmem30a	0.37986667	0.33252972
Acyl-CoA-binding protein	Dbi	0.36446667	0.07094692
Annexin A5	Anxa5	0.32246667	0.13071344
Syntaxin-2	Stx2	0.3114	0.09850358
Ras-related protein Rab-5C	Rab5c	0.28773333	0.12020886
General vesicular transport factor p115	Uso1	0.28533333	0.41073496
Ras-related protein Rab-8A	Rab8a	0.2654	0.17811217
Endophilin-B1	Sh3glb1	0.23896667	0.64886981
Ras-related protein Rab-6A	Rab6a	0.2291	0.34524828
COP9 signalosome complex subunit 5	Cops5	0.2267	0.19568233
Heat shock cognate 71 kDa protein	Hspa8	0.2044	0.28574623
Ras-related protein Rab-10	Rab10	0.12693333	0.36351494
Ras-related protein Rab-11B	Rab11b	0.12416667	0.32150213
AP-1 complex subunit gamma-1	Ap1g1	0.10696667	0.78671066
Ras-related protein Rab-35	Rab35	0.1055	0.69913638
V-type proton ATPase subunit d 1	Atp6v0d1	0.07626667	0.89081126
Syntaxin-3	Stx3	0.06873333	0.72051544
Peflin	Pef1	0.03376667	0.89760559
Transmembrane emp24 domain-containing protein 10	Tmed10	−0.0147667	0.92350375
Ras-related protein Rab-5A	Rab5a	−0.0152	0.8959618
DnaJ homolog subfamily C member 5	Dnajc5	−0.0513667	0.69111549
Protein SEC13 homolog	Sec13	−0.0656667	0.64568037
Beta-2-syntrophin	Sntb2	−0.132	0.29493827
Calcineurin B homologous protein 1	Chp1	−0.1357667	0.17958119
Dynamin-1-like protein	Dnm1l	−0.1607333	0.63163875
Ras-related protein Rab-8B	Rab8b	−0.2352667	0.4343227
Ras-related protein Rab-14	Rab14	−0.2371667	0.13506351
Protein transport protein Sec23A	Sec23a	−0.2438	0.735827
Protein transport protein Sec31A	Sec31a	−0.2536333	0.10230817
Syntaxin-12	Stx12	−0.2754667	0.13695112
Phosphatidylinositol-binding clathrin assembly protein	Picalm	−0.3006333	0.73843355
SEC23-interacting protein	Sec23ip	−0.3045667	0.32428489
GTP-binding protein SAR1a	Sar1a	−0.3352	0.11837466
Secretory carrier-associated membrane protein 1	Scamp1	−0.3650333	0.54405532
Voltage-dependent anion-selective channel protein 1	Vdac1	−0.4649667	0.00746836
Copper-transporting ATPase 1	Atp7a	−0.5209667	0.19120787
V-type proton ATPase 116 kDa subunit a isoform 1	Atp6v0a1	−0.5223	0.49067594
Voltage-dependent anion-selective channel protein 2	Vdac2	−0.5464667	0.01837449
Programmed cell death protein 6	Pdcd6	−0.5767333	0.07925068
Vesicle-associated membrane protein 7	Vamp7	−0.5904333	0.67137801
Voltage-dependent anion-selective channel protein 3	Vdac3	−0.6144333	0.31277254
Cation-independent mannose-6-phosphate receptor	Igf2r	−0.7044	0.0856667
Protein transport protein Sec23B	Sec23b	−0.787	0.00603393
Myc box-dependent-interacting protein 1	Bin1	−0.8680667	0.25621378
Secretory carrier-associated membrane protein 2	Scamp2	−0.8799333	0.5918047
Vesicle-trafficking protein SEC22b	Sec22b	−1.0781667	0.01000553
Biglycan	Bgn	−1.1482	0.30109161

### Comparison of Human Plasma EVs Isolation Methods for Proteomics

**FIGURE 6:**
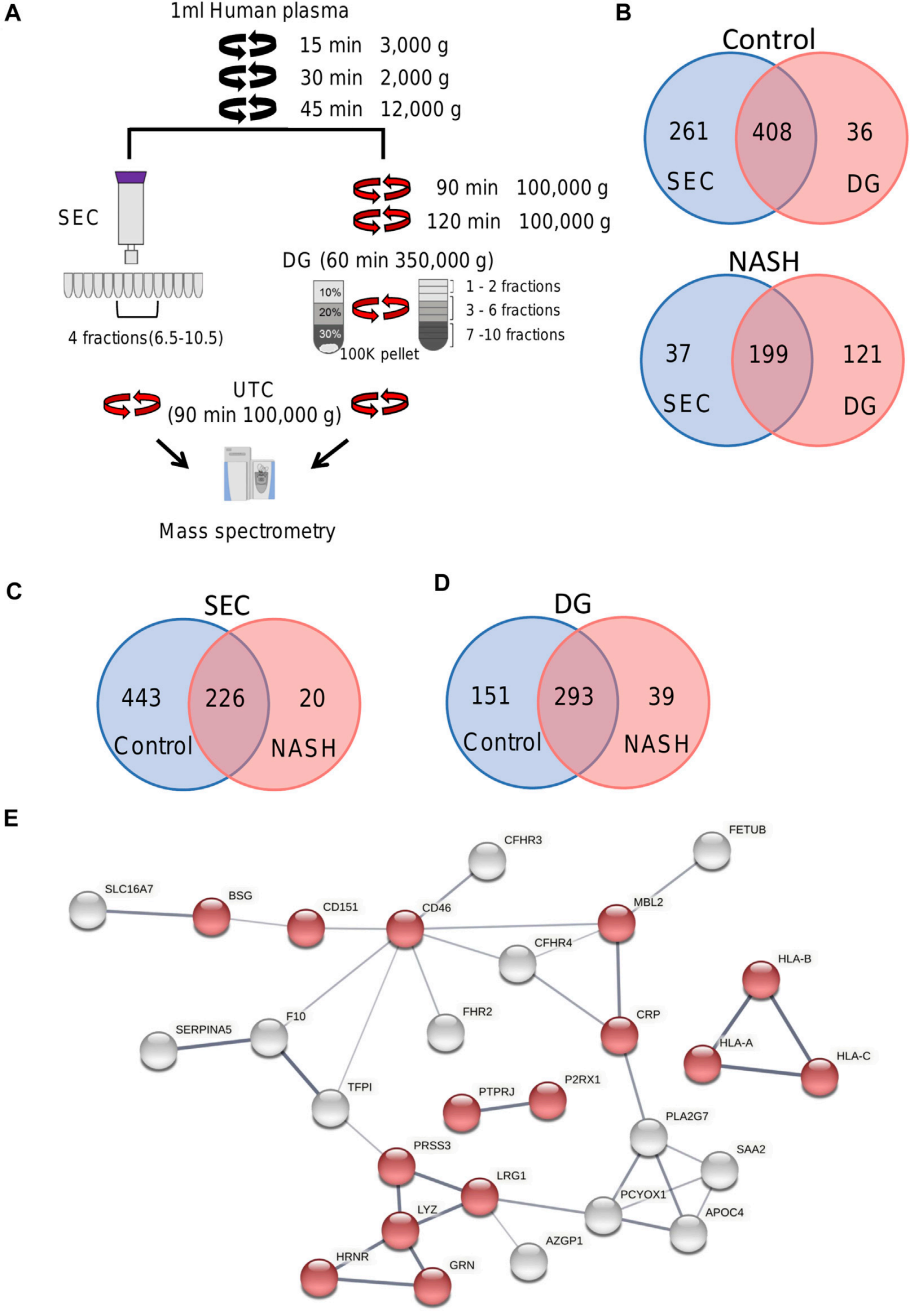
Overview of plasma EV proteomics methods. **(A)** Schema represents two different EV isolation methods. In SEC, fractions 6.5 to 10.5 were combined and pelleted by UTC. For DG fractions 1–2, 3–6, and 7–10 were collected and combined. **(B)** Venn diagram depicting the number of common and unique proteins in control and NASH plasma EVs isolated by SEC compared to DG fractions 3–6. **(C)** Venn diagram depicting the number of common and unique proteins in control plasma EVs isolated by SEC and NASH plasma EVs isolated by SEC. **(D)** Venn diagram depicting the number of common and unique proteins in control plasma EVs isolated by DG fractions 3–6 and NASH plasma EVs isolated by DG fractions 3–6. **(E)** Protein–protein interaction analysis was performed by STRING software on unique NASH EV proteins; the edges indicate both functional and physical protein associations; line thickness indicates the strength of data support; we used minimum required interaction score with medium confidence (0.400); disconnected nodes in the network were excluded; red color represents immune system process. The annotation of these proteins was done by using Gene Ontology.

**FIGURE 7:**
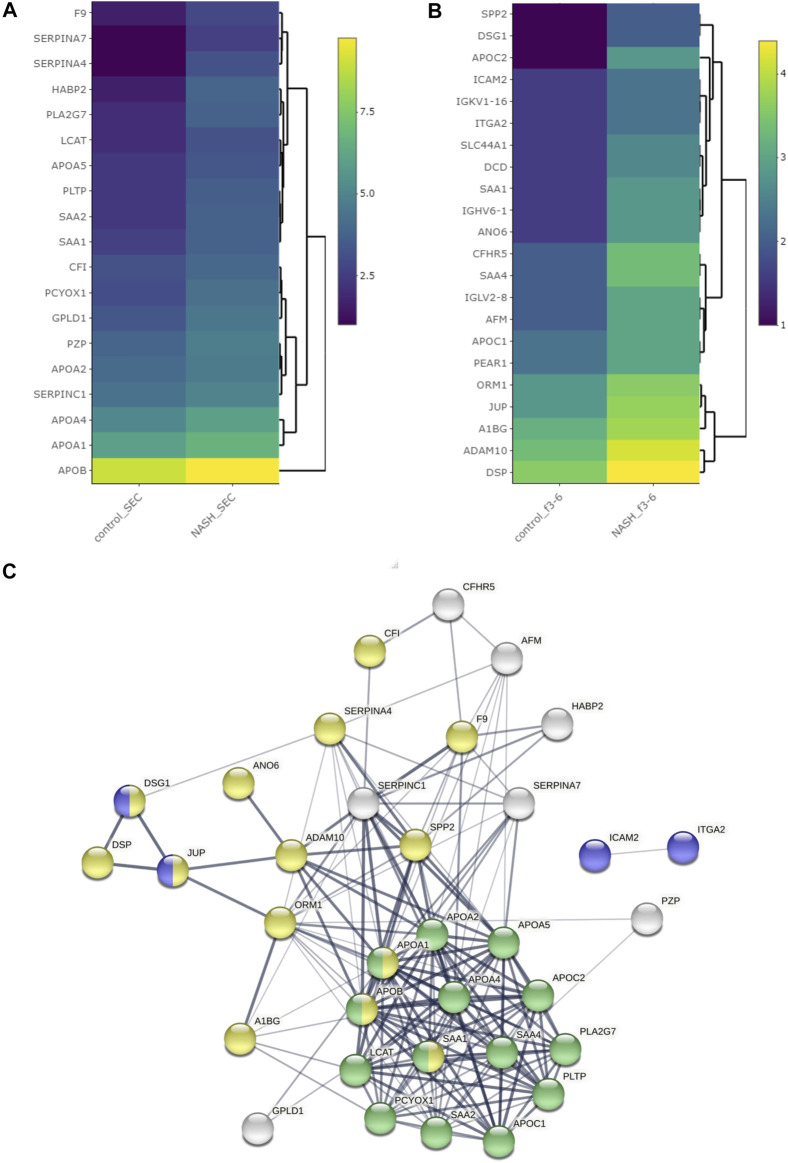
Differentially expressed proteins among plasma EVs. Heatmap representing proteins with greater than 1.5-fold change in NASH plasma EVs, which were isolated **(A)** by SEC and **(B)** by DG. **(C)** Protein–protein interaction analysis was performed by STRING software on significantly enriched proteins in NASH EVs; the edges indicate both functional and physical protein associations; line thickness indicates the strength of data support; we used minimum required interaction score with medium confidence (0.400); disconnected nodes in the network were excluded; blue color represents cell adhesion proteins; green color represents plasma lipoprotein particle protein; yellow color represents vesicle-mediated transport; these proteins annotation was done by using Gene Ontology and UniProt.

We isolated EVs from plasma from a healthy control and a pooled NASH sample. Each plasma sample was divided into two 1-ml aliquots and EVs were isolated by SEC or DG (Figure 6A). We collected and combined fractions 1–2, 3–6, and 7–10 obtained by DG. EVs were higher in NASH plasma than control by each isolation method (Supplementary Figure S5A,B). Proteomic analysis of EV fractions isolated from DG fractions (Supplementary Figure S5C) demonstrated that fractions 3–6 had the highest numbers of proteins. Furthermore, comparison of cellular components by GO analysis demonstrated that fractions 3–6 had the highest ratio of exosome proteins and plasma membrane proteins and the lowest ratios of blood microparticle proteins, high-density lipoprotein particle, and very-low-density lipoprotein particle (Supplementary Figure S5D), demonstrating that fractions 3–6 had least contribution from platelet-derived microvesicles and lipoprotein particles. A greater number of EV proteins was isolated by SEC than DG (sum of all fractions) in control EV (Figures 6B,D). The number of NASH unique proteins isolated by SEC and DG fractions 3–6 were 20 and 39, respectively (Figures 6C,D). We performed STRING analysis by using these NASH unique proteins (Figure 6E). Included among these proteins were immune system processing proteins such as MHC class 1 proteins and C-reactive protein. We next examined the expression levels of proteins between control and NASH, which were detected in both conditions to determine changes in expression. Figure 7A represents more than 1.5-fold increase in EV proteins in NASH isolated by SEC. The highest fold change protein was hyaluronan-binding protein 2 (HABP2). Figure 7B represents more than 1.5-fold increased EV proteins in NASH isolated by DG. In the comparison of NASH EV proteins to control EV proteins, the three proteins with greatest differential expression (fold change) were serum amyloid A-1 protein (SAA1, 2.3-fold), immunoglobulin heavy variable 6-1 (IGHV6-1, 2.3-fold), and anoctamin-6 (ANO6, 2.3-fold), though these were not the most abundant. Lastly, we combined these NASH expressed proteins either isolated by SEC or DG and performed STRING analysis (Figure 7C). This analysis revealed that these proteins contain adhesion proteins, vesicle-mediated transport proteins, and apolipoproteins.

### Biological Function Analysis of Hepatocyte-Derived EVs

**FIGURE 8:**
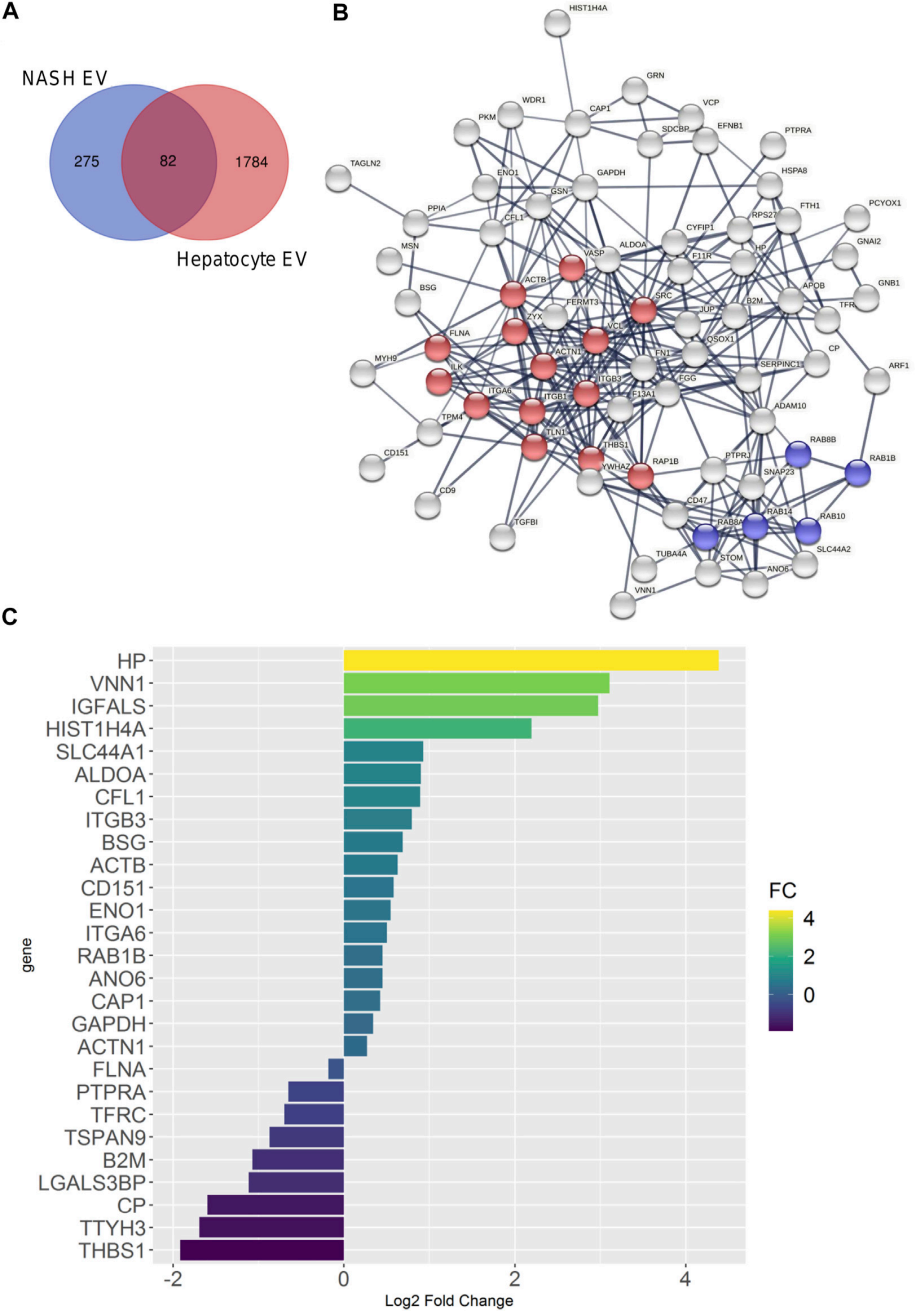
Comparison of palmitate-stimulated EV proteins with NASH plasma EV proteins. **(A)** Venn diagram compared 357 proteins of human NASH plasma EVs and 1,866 proteins of mouse hepatocyte-derived, palmitate-stimulated EVs. **(B)** Protein–protein interaction analysis was performed by STRING software; the edges indicate both functional and physical protein associations; line thickness indicates the strength of data support; we used minimum required interaction score with high confidence (0.700); disconnected nodes in the network were excluded; red color represents focal adhesion proteins; blue color represents RAB subfamily of small GTPases proteins; these proteins annotation was done by using KEGG and SMART. **(C)** Bar graph depicts EV proteins detected in ceramide-dependent palmitate-stimulated lipotoxic EVs and NASH plasma EVs; proteins are shown which had *p* < 0.05; *x*-axis and color represents Log2 fold change.

Plasma EVs are derived from multiple cellular sources. We employed the more homogeneous hepatocyte-derived EV proteins to determine whether hepatocyte-derived EVs were detected in the more heterogenous plasma EV proteins. In this analysis, we included 357 proteins detected in human NASH plasma EVs (by SEC and DG) and 1,866 proteins detected in hepatocyte-derived palmitate-stimulated EVs. There were 82 conserved proteins between both conditions (Figure 8A). Figure 8B demonstrates the STRING analysis of 82 conserved proteins. Included were focal adhesion proteins such as integrin and Rab subfamily of small GTPases (RAB1B, RAB8A, RAB8B, RAB10, and RAB14). Among 82 proteins, coagulation factor XIII A chain (F13A1) was significantly increased (6.1-fold change) by palmitate stimulation. Figure 8C depicts EV proteins detected in NASH plasma EVs and ceramide-dependent palmitate-stimulated EVs that were significantly upregulated or downregulated. The three EV proteins that were most enriched in lipotoxic EVs in a STARD11-dependent manner (Table 5) and detected in the human NASH plasma sample were haptoglobin, VNN1, and IGFALS.

**TABLE 5:** Palmitate-stimulated EV proteins also detected in plasma.

Protein description	Gene name	Log2 FC WTVC and STARD11-/-VC	*p*-value	Log2 FC WTPA and STARD11-/-PA	*p*-value
Haptoglobin	Hp	1.71966667	0.328	4.38716667	0.000993
Pantetheinase	Vnn1	2.7149	0.000382	3.10726667	0.000682
Insulin-like growth factor-binding protein complex acid labile subunit	Igfals	−0.5061667	0.636	2.97173333	0.0464

**FIGURE 9:**
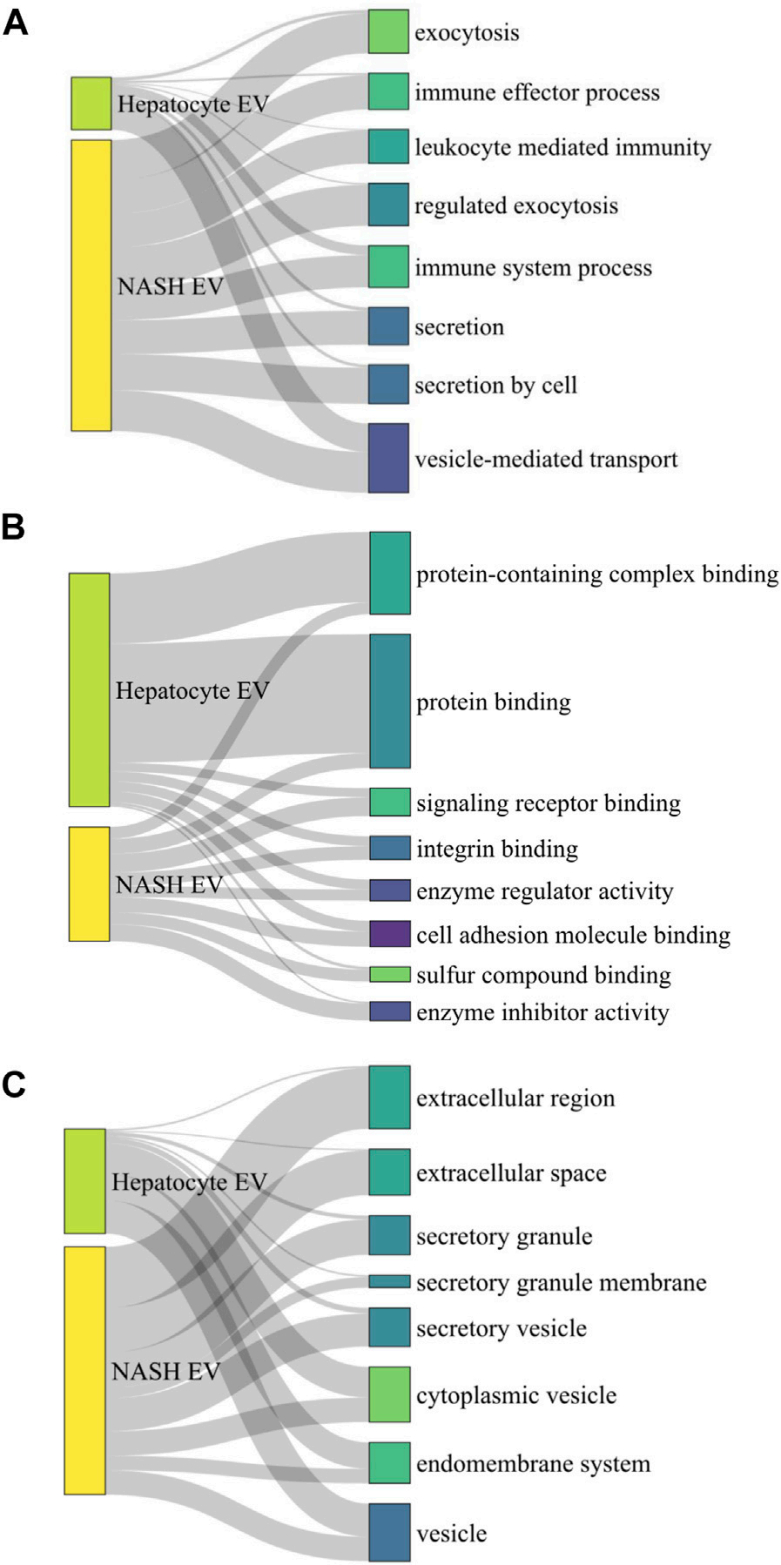
Sankey diagram linking palmitate-stimulated EV proteins with NASH plasma EV proteins. Sankey diagram linking top 8 commonly detected gene ontology (GO) terms from 1,766 hepatocyte-derived palmitate-stimulated EV proteins and 357 human NASH plasma EV proteins, **(A)** by biological process analysis, **(B)** by molecular function analysis, and **(C)** by cellular component analysis. A ribbon’s thickness indicates −log10FDR for each cluster of GO terms in each GO analysis.

Next, we performed comparison of GO analysis including biological process, molecular function, and cellular component. The numbers of common GO terms annotated as biological process, molecular function, and cellular component were 450, 64, and 86, respectively. Among these common GO terms, we selected top 8 ordered by FDR and depict them as Sankey diagrams in which the arrow width is proportional to the expression level. In biological process analysis, vesicle-mediated transport had the highest FDR ratio between hepatocyte-derived lipotoxic EVs and NASH plasma EVs (Figure 9A). Immune system processes, leukocyte-mediated immunity, and immune effector processes were also conserved between the two types of EVs, in keeping with the known proinflammatory role of hepatocyte-derived lipotoxic EVs (Kakazu et al., 2016; Dasgupta et al., 2020). Molecular function analysis demonstrated that hepatocyte-derived EVs were enriched in protein-containing complex binding, protein binding, signaling receptor binding, and integrin binding proteins (Figure 9B), all of which are also consistent with the known mechanisms by which EVs activate effector cell responses (Zhou et al., 2020). Lastly, in cellular component analysis, we confirmed that there are EV-related GO terms in both hepatocyte-derived and NASH plasma EVs (Figure 9C).

## Discussion

EVs are being widely appreciated for their role in cell-to-cell communication and as important mediators in multiple biological functions in NASH pathogenesis, which are mediated by unique EV cargoes. To investigate the proteomic cargoes of EVs, we compared EV isolation methods. Additionally, employing optimized EV proteomics methods, we investigated ceramide trafficking-dependent lipotoxic EV cargoes and compared these EVs with NASH plasma EVs to determine conserved EV-derived signaling pathways. Our principal findings are as follows: 1) SEC yielded the most number of proteins, while preserving the top 100 EV proteins and select EV marker proteins in comparison to UTC and DG; 2) lipotoxic EVs are enriched in many distinct DAMPs and ribosomal proteins; 3) ICAM1 and PRKCA were the most significant ceramide trafficking-dependent lipotoxic EV proteins; and 4) F13A1, haptoglobin, VNN1, and IGFALS were detected in lipotoxic hepatocyte-derived EVs and in NASH plasma EVs, providing candidates for further validation studies.

Efficient isolation of EVs has been an active area of research to understand their biological properties and to explore their potential as disease biomarkers (Melo et al., 2015; Allenson et al., 2017; Palmirotta et al., 2018). Over the past decade, UTC has been the most utilized method for EV isolation (Chhoy et al., 2021). On the other hand, there are a variety of EV isolation methods based on biophysical properties or targeted epitope-based capture (Kowal et al., 2016; Patel et al., 2019). However, the optimal isolation methods for cell culture and plasma EV proteomics remain unclear. Plasma EV isolation is also accompanied with the risks of co-isolating plasma proteins and lipoprotein particles. Thus, any EV isolation method for proteomics analysis must balance yield with purity. Here, we compared SEC, UTC, and DG methods. All the methods were applied to isolate small EVs from the supernatant of 10,000 × g spin to eliminate large EVs. In the comparison of cell culture EV proteomics, SEC yielded the most ExoCarta annotated proteins from both species and abundance aspect. The top 100 ExoCarta EV proteins were almost all identified in EVs isolated by SEC, and SEC identified the most proteins of all three methods. Thus, SEC provided the sensitivity needed for novel cargo discovery while preserving specificity. Therefore, based on yield, abundance, and detection of the top 100 ExoCarta proteins, we selected SEC as the cell culture EV proteomics method for further analyses of ceramide-dependent palmitate-stimulated EV proteome.

Proteomic analysis of lipotoxic EVs detected S100 family proteins and annexin family proteins among the most abundant proteins in lipotoxic EVs. Cellular responses to stress include the release of a group of S100 proteins that function as DAMPs (Xia et al., 2018; Sedaghat and Notopoulos, 2008). Recent studies have demonstrated that S100A11 is an inducible hepatocyte DAMPs (Sobolewski et al., 2020; Zhang et al., 2021). In our data, S100A11 was enriched in EVs as a ceramide trafficking-dependent lipotoxic EV cargo, suggesting that S100A11 may play a role in NASH. Furthermore, it is well known that annexin family proteins are commonly found in EVs (Simpson et al., 2012). It is suggested that the annexin family proteins localize to EVs due to their membrane trafficking function and may play a role in cargo selection into EVs (Popa et al., 2018). As shown in STRING analysis (Figure 4B), both S100 and annexin family proteins are known to bind together (Rintala-Dempsey et al., 2008). The interactions of S100–annexin complexes may associate these DAMPs with lipotoxic EVs (Ibrahim et al., 2018).

In the top 100 lipotoxic EV proteins, we detected eukaryotic translation elongation factor 1A-1 (eEF1A-1), which is known to be induced in hepatocytes downstream of lipotoxic ER stress (Stoianov et al., 2015). It is reported that eEF1A1 inhibition reduces lipotoxicity in obese mice with NAFLD (Hetherington et al., 2016; Wilson et al., 2020). The canonical function of eEF1A1 is to recruit aminoacyl-tRNAs to the ribosome during peptide elongation (Mateyak and Kinzy, 2010). In addition to eEF1A1, we detected numerous ribosomal proteins in lipotoxic EVs. The GO biological process analysis suggested that the proteins involved in translation, RNA processing, ribosome biogenesis, and proteolysis processes were enriched in the lipotoxic EVs (Supplementary Figure S3). The mechanism for an increase in ribosomal proteins in EVs is likely related to palmitate-induced lipotoxic ER stress. The upregulation of ribosomal proteins under ER stress conditions is known (Mandal et al., 2016). These data suggest that the proteome of EV released from hepatocytes undergoing lipotoxic ER stress reflects the state of cell of origin. In keeping with our findings, Zhu et al. found the components necessary for translation all existed in macrophage exosomes, including mRNA, tRNA, ribosomes, and tRNA-ligase, and hypothesized that EVs might independently express specific proteins (Zhu et al., 2015). Ribosomal protein (RP) L36A and RPL14 were reported in NAFLD and NASH human liver gene expression data (Wang et al., 2016). It is further reported that loss of small nucleolar RNAs (snoRNAs) encoded in the RPL13A is sufficient to confer resistance to lipotoxic and oxidative stress (Michel et al., 2011). In our dataset, we observed high expression of RPL13A in lipotoxic EVs. EV-mediated snoRNA transfer could enhance metabolic efficiency in recipient cells (Rimer et al., 2018). We also detected nucleophosmin (NPM1), which is involved in several cellular processes, including centrosome duplication, protein chaperoning, and cell proliferation. These RNA binding proteins (RBPs) could serve as key players in this mechanism, by making complexes with RNAs and transporting them into EVs during the biosynthesis of EVs (Statello et al., 2018).

In our ceramide-dependent lipotoxic EV analysis, ICAM1 was highly upregulated. Rat hepatocyte-derived EV proteomics (Conde-Vancells et al., 2008) and NAFLD mouse model EV detected ICAM1 (Povero et al., 2014b). The plasma membrane colocalization and interaction of ICAM1 and ceramide was reported during endocytosis (Serrano et al., 2012), suggesting that ICAM1 and ceramide might be involved in the uptake of lipotoxic EVs by target cells. Another protein enriched in a ceramide-dependent manner on lipotoxic EVs protein is protein kinase C-α, which can be directly activated by ceramide (Müller et al., 1995; Huwiler et al., 1998; Aschrafi et al., 2003), and is induced in the liver in NASH (Wang et al., 2016). These proteins might serve as ceramide-dependent lipotoxic EV markers and suggest that EV cargo is influenced by the signaling pathways known to be upregulated in lipotoxicity.

Next, we compared EV proteomics from human plasma EVs isolated by SEC or DG. We excluded UTC from human plasma EV proteomics to avoid confounding by contaminating lipoprotein particles and protein aggregates. In NASH plasma EVs, both methods yielded several enriched EV proteins. Among these, serum amyloid A1 (SAA1) was twofold higher than control plasma EVs in both methods. SAA1, SAA2, and SAA4 were also enriched in NASH EVs. It is reported that patients with active liver diseases including NASH had higher serum SAA levels than healthy controls (Yuan et al., 2019). SAA proteins can interact with cell surface receptors and integrins, suggesting a mechanism by which these proteins may be a constituent of EV cargo. ICAM2 was also higher in NASH EV protein isolated by DG. It is reported that ICAM2 on human EVs proteomics can differentiate healthy controls from patients with pre-cirrhotic and cirrhotic NASH (Povero et al., 2020). Immune system process proteins such as C-reactive protein (CRP) and MHC class 1 proteins were detected among 53 NASH unique proteins.

Plasma EVs arise from heterogenous cellular sources; therefore, to determine if hepatocyte-derived EVs are indeed a component of plasma EVs, we compared the proteome of lipotoxic hepatocyte-derived EVs and NASH plasma EVs. Among the proteins that were detected in both sample sets, coagulation factor XIII A Chain (F13A1) was upregulated the most in palmitate-stimulated lipotoxic EVs. This protein was also in plasma EVs isolated from a dietary NASH mouse model (Povero et al., 2014a) and its gene expression was reported to correlate with fat content in human NAFLD (Greco et al., 2008). The top three ceramide-dependent lipotoxic EV proteins were haptoglobin, VNN1, and IGFALS. Haptoglobin is an acute phase protein mainly produced by hepatocytes and detected in plasma EVs in a murine NASH model (Giffen et al., 2003; D’souza et al., 2012). Serum fucosylated-haptoglobin level was reported as a potential diagnostic biomarker for NASH (Kamada et al., 2013). Hepatic VNN1 expression and activity were previously shown to be significantly induced by dietary fatty acids (Rakhshandehroo et al., 2010). Hepatocyte-derived lipotoxic EVs enriched in VNN1 are internalized into endothelial cells and hepatic stellate cells in NASH (Povero et al., 2013; Povero et al., 2015). IGFALS is reported to be upregulated in NASH patients (Younossi et al., 2005). This comparative analysis suggests that F13A1, haptoglobin, VNN1, and IGFALS are potential markers to detect hepatocyte-derived lipotoxic EVs in plasma and serve as biomarkers for NASH. However, these observations will need to be further validated.

In the GO analysis of biological function, EV characteristics, immune effector processes, leukocyte-mediated immunity, and immune system processes were commonly represented in hepatocyte EVs and NASH EVs. NASH is characterized by chronic sterile inflammation in which hepatocyte-to-immune cell communication plays a key role (Hirsova et al., 2016b). Thus, the conserved immune system processes are consistent with the known and emerging role of hepatocyte-derived EVs in recruiting proinflammatory monocytes into the liver directly or *via* sinusoidal endothelial cells. Hepatocyte-derived lipotoxic EVs may also activate hepatic stellate cells (Povero et al., 2015). In the context of EV-mediated intercellular communication in NASH, we and others have characterized hepatocyte-EV derived recipient cell responses for individual bio-active cargoes (Hirsova et al., 2016a; Liao et al., 2018; Furuta et al., 2021). It is also important to note that EVs contain complex cargoes with nuclei acids, lipids, and protein ligands. Signaling roles for some of these cargoes in NASH include microRNAs (miR-128-3p), lipids (sphingosine 1-phosphate), and proteins (CXCL10, Vanin) (Povero et al., 2013; Povero et al., 2015; Ibrahim et al., 2016; Liao et al., 2018). How these complex cargoes interact in recipient cell responses remains to be experimentally tested; for example, a balance of changes in proinflammatory versus anti-inflammatory cargoes per EV or proinflammatory EV concentration versus anti-inflammatory EV concentration may determine recipient cell responses. Our human dataset is a significant limitation due to the small sample size. These findings will need confirmation in larger human plasma EV proteomic datasets. Here, our objective with the human plasma samples was to optimize and disseminate our comparative methods for plasma EV proteomics.

In summary, we have provided a methodological resource for investigators and defined ceramide-dependent proteomic cargo of EVs. Hepatocyte-derived lipotoxic EVs contain DAMPs and cell adhesion molecules such as S100a11 and ICAM1, which might affect immune cell responses, thus promoting liver inflammation in NASH. Limited analysis of human NASH EV proteome included ICAM2, F13A1, haptoglobin, VNN1, and IGFALS, which were also detected in hepatocyte-derived EVs. These findings will need further validation in larger plasma datasets and as technological advances permit the identification of hepatocyte-derived EVs in plasma with minimal processing.

## Data Availability

The datasets presented in this study can be found in online repositories. The names of the repository/repositories and accession number(s) can be found below: http://proteomecentral.proteomexchange.org/ PXD028175.

## Glossary

ANGPTL2: angiopoietin-like protein 2
ANO6: anoctamin-6
COL1A1: collagen alpha-1 chain
DAMP: damage-associated molecular pattern
DG: density gradient
Emb: embigin
ER: endoplasmic reticulum
EV: extracellular vesicles
FDR: false discovery rate
GO: gene ontology
Hp: haptoglobin
H3F3A: histone H3.3
ICAM1: intercellular adhesion molecule 1
IGFALS: insulin-like growth factor-binding protein complex acid labile submit
IGHV6-1: immunoglobulin heavy variable 6-1
IGSF3: immunoglobulin superfamily member 3
IMH: immortalized mouse hepatocytes
IRE1α: inositol requiring enzyme 1 alpha
MHC: major histocompatibility complex
NTA: nano-particle tracking analysis
NAFLD: non-alcoholic fatty liver disease
NASH: non-alcoholic steatohepatitis
PALM3: paralemmin-3
PTK7: protein tyrosine kinase 7
PRKCA: protein kinase C alpha type
SEC: size exclusion chromatography
SAA1: serum amyloid A-1 protein
STARD11: StAR-related lipid transfer domain 11
tSNE: t-distributed stochastic neighbor embedding
UTC: ultracentrifugation
VNN1: vanin 1
WNT10A: wnt family member 10A
