# Supplementary material for: A Comparative Proteomic Analysis of Extracellular Vesicles Associated With Lipotoxicity
Source: Front Cell Dev Biol. 2021 Nov 4;9:735001. doi: 10.3389/fcell.2021.735001 (PMC8600144; doi:10.3389/fcell.2021.735001)
Supplement: Supplementary file 1 [file DataSheet1.DOCX]

Supplementary Material

**A comparative proteomic analysis of extracellular vesicles associated with lipotoxicity**

**Yasuhiko Nakao^1,2^; Masanori Fukushima^1,2^; Amy S. Mauer^1^; Chieh-Yu Liao^1^; Anya Ferris^1,3^; Debanjali Dasgupta^1,4^; Carrie Jo Heppelmann^5^, Patrick Vanderboom^5,6^, Mayank Saraswat^7,8,9^; Akhilesh Pandey^7,8,9,10^; K Sreekumaran Nair^6^; Alina M. Allen^1^; Kazuhiko Nakao^2^; Harmeet Malhi^1*^**

##
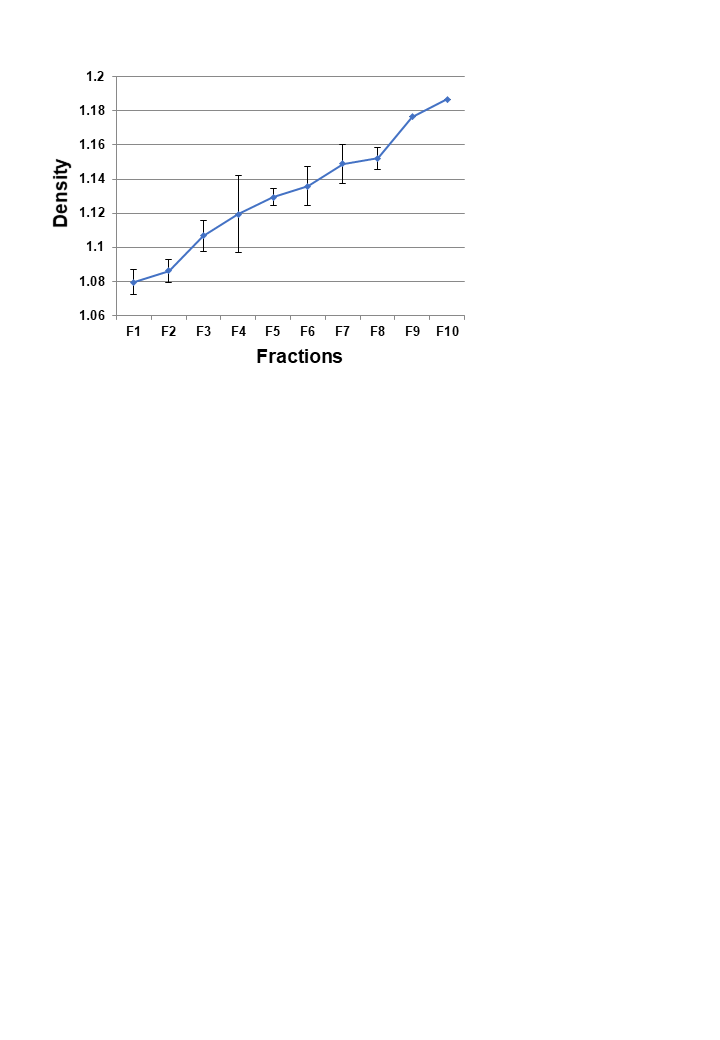


**Supplementary Figure 1.** **Density of iodixanol gradient fractions**. The density of each collected fraction by iodixanol density gradient was determined.


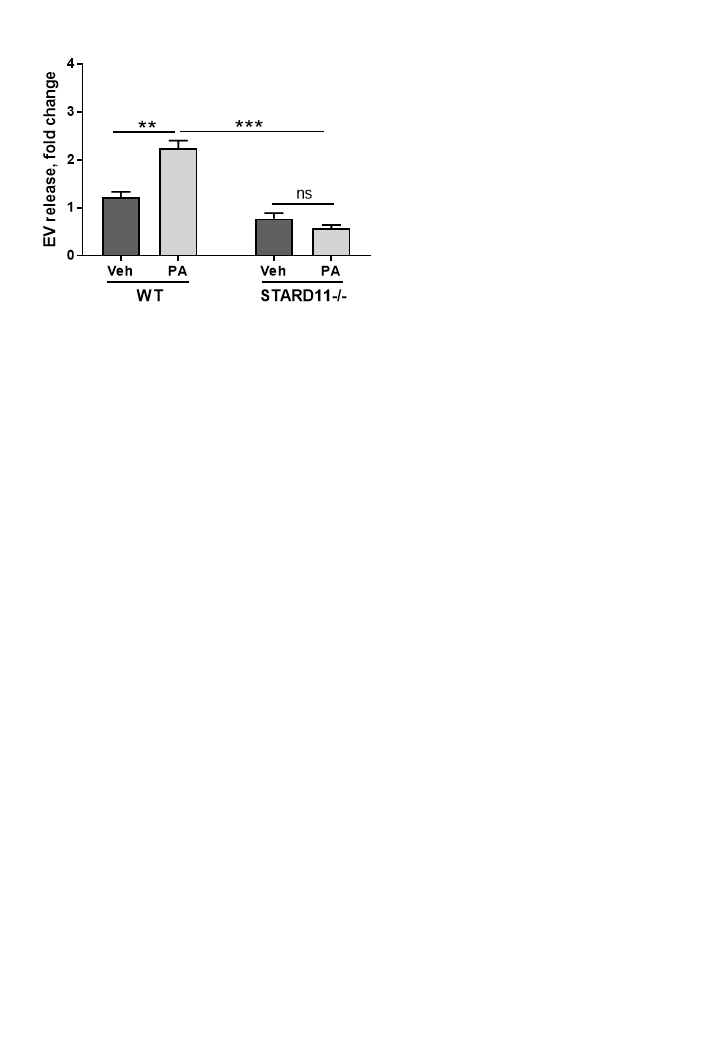


**Supplementary Figure 2.** **Lipotoxic EV release is STARD11 dependent.** Wildtype (WT) and STARD11 knockout (STARD11-/-) cells were treated with palmitate (400 µM, 16 hours) and EVs isolated by size exclusion chromatography, fractions 6.5-10.5 were combined, pelleted by ultracentrifugation, and quantified by nanoparticle tracking analysis. N=3, **p<0.01, ***p<0.001.

**Supplementary Figure 3.** **Number of proteins in STARD11-/- and WT EVs**. The Venn diagram depicts the number of proteins identified in EVs from WT or STARD11-/- cells treated with palmitate (PA) or vehicle (Veh). Uniquely and commonly detected proteins are indicated in the diagram.


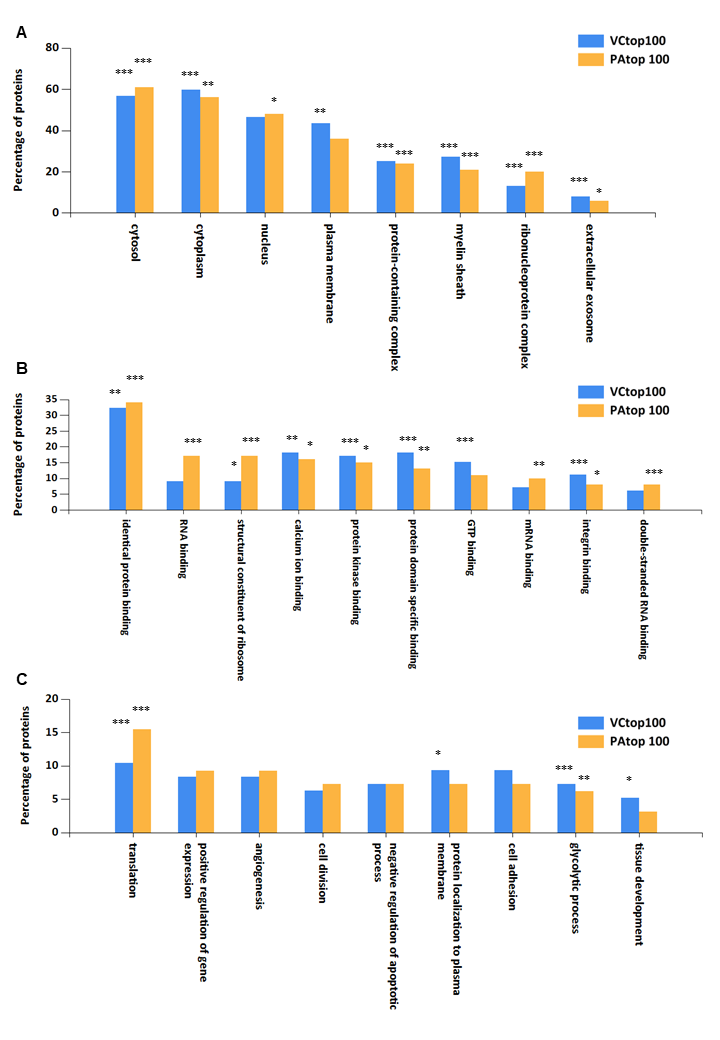


**Supplementary Figure 4.** **Gene ontology analysis of most abundant EV proteins.** The bar graphs show gene ontology about A) cellular component, B) molecular function, and C) biological process. In each graph, the y-axis represents percentage of proteins; blue bars represent top100 expressed proteins in Vehicle EV (VCtop100) and orange bar represents top 100 expressed proteins in PA EV (PAtop100). FDR was calculated by Benjamini-Hochberg procedure. FDR values of <0.05, 0.01, and 0.001 were denoted as *, **, and *** respectively.


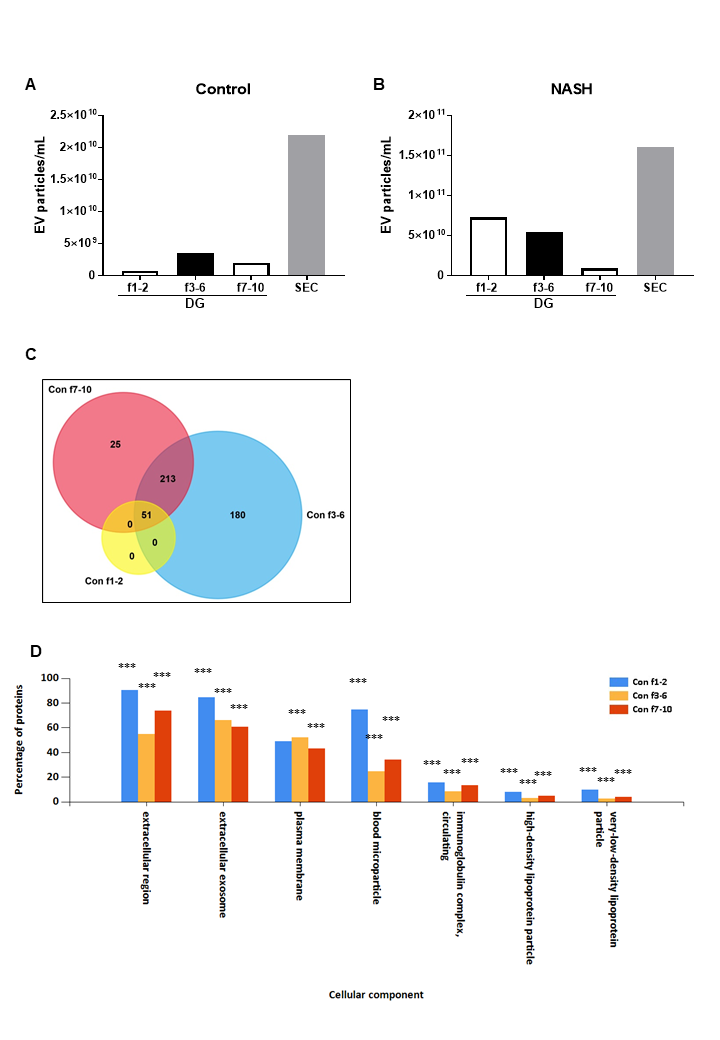


**Supplementary Figure 5. Analysis of plasma EVs and EV proteins.** A and B) Circulating EVs were measured in 1 mL of plasma from control and NASH samples. C) Venn diagram shows number of unique proteins among 3 different DG fractions from control. D) Bar graph shows gene ontology analysis about cellular component; y-axis represents percentage of proteins; blue bar represents EV proteins isolated from DG fraction 1-2; orange bar represents EV proteins isolated from DG fraction 3-6; red bar represents EV proteins isolated from DG fraction 7-10. FDR was calculated by Benjamini-Hochberg procedure. FDR values of <0.05, 0.01, and 0.001 were denoted as *, **, and *** respectively.
